# Metal Fractionation in Surface Sediments of the Brahmaputra River and Implications for Their Mobilization

**DOI:** 10.3390/ijerph17249214

**Published:** 2020-12-09

**Authors:** Tenzin Tsering, Mika Sillanpää, Satu-Pia Reinikainen, Mahmoud S. M. Abdel Wahed

**Affiliations:** 1 LUT School of Engineering Sciences, Lappeenranta University of Technology, P.O. Box 20, FI-53851 Lappeenranta, Finland; Satu-Pia.Reinikainen@lut.fi; 2 Institute of Research and Development, Duy Tan University, Da Nang 550000, Vietnam; 3 Faculty of Environment and Chemical Engineering, Duy Tan University, Da Nang 550000, Vietnam; 4 School of Civil Engineering and Surveying, Faculty of Health, Engineering and Sciences, University of Southern Queensland, West Street, Toowoomba, QLD 4350, Australia; 5 Department of Chemical Engineering, School of Mining, Metallurgy and Chemical Engineering, University of Johannesburg, P.O. Box 17011, Doornfontein 2028, South Africa; 6 Department of Geology, Faculty of Science, Beni-Suef University, Beni-Suef 62521, Egypt; mahmoud.abdelwahed@science.bsu.edu.eg

**Keywords:** Brahmaputra River, heavy metal, sequential extraction, sediments, turbidity, India

## Abstract

The Brahmaputra River is the largest tropical river in India that flows along the Himalayan regions and it is the lifeline of millions of people. Metal fractionation in the Brahmaputra River’s surface sediments and its correlation with turbidity are assessed in this study. The interaction between metal fractions and the overlying water is studied using multivariate statistical analyses. The strong positive correlation between NH_4_ of the overlying water and the exchangeable fractions in sediments signifies that the metals in the exchangeable fractions can be substituted by NH_4._ Subsequently, these metals can be released into the overlying water. The fluctuation in turbidity from 73 to 875 NTU indicates a large variation in the suspended matter concentration, and a higher concentration of suspended matter could provide attachment sites for pollutants such as metals. Significant variation in turbidity manifests a potentially high risk of pollution. In addition, the observation of local people along the Brahmaputra River turning its color to muddy indicates the need for continuous monitoring of water quality and an assessment of pollution is crucial. Although the Brahmaputra River’s risk assessment code is at low risk, the exchangeable fractions of Ni and Zn are present at all sites. Thus, the Brahmaputra River requires early preventive measures and management strategies to control metal pollution. This study contributes to an understanding of the fluctuation of turbidity of a tropical river. We provide baseline data for policymakers, and the importance of further intensive studies on metal pollution in the Himalayan Rivers is highlighted.

## 1. Introduction

Over 90% of inorganic pollutants can be trapped in the sediments of the aquatic system during hydrological cycles, and metals are major contaminants [1,2]. The remaining <10% of inorganic pollutants can be trapped in water, soil and atmosphere. Metals are input from natural sources and anthropogenic activities [3,4,5,6]. The persistence, abundance, toxic nature of metals and their subsequent bioaccumulation pose a serious risk to the aquatic ecosystem.

Nemati et al. [7] investigated the distribution pattern of metal species in different depths of sediment and indicated that the enrichment of the metals at the topmost layer of sediment showed the pollution from recent years and could be deposited from the surface water. Levels of metal pollution are controlled by granulometric characteristics of sediments, as Borah et al. [8] indicated that absorption of metals (Zinc, Arsenic, Copper) increases with the increase in area of the sediment, and Vosoogh et al. [9,10] observed that concentration of metals (Copper, Vanadium, Cadmium, Chromium) was higher in sediment sizes larger than 63 µm. Kang et al. [5] studied the effect of dissolved oxygen and nutrient level (NH_4_^+^, NO_3_^−^ and PO_4_^3−^) of the overlying water on the metal speciation and metal contents in river surface sediment. Fractionation of metal in the sediment may be affected by bioturbation, organic decay and change in pH and oxidation reduction potential (ORP) of the overlying water [5,7,11,12,13,14,15]. To understand chemical fractionation of heavy metals, the four-step sequential extraction procedure proposed by the Community Bureau of Reference (BCR) has been significantly applied in many studies due to the method validation and quality control by using certified reference materials such as BCR-601, BCR-701 and BCR-483 [16].

Fractionation of the metals in sediment of the tropical rivers such as the Brahmaputra River is significant but rarely studied [8,17]. Pallavi et al. [17] investigated the high risk of Cu and Pb in the Brahmaputra River sediment but only studied the Assam region. The Brahmaputra River is the largest tropical river in India by water discharge [18,19]. The residents along the Brahmaputra River flow in Arunachal Pradesh and Assam are witnessing the highly muddy color of the overlying water that was an unusual phenomenon before [20,21]. In this light, the objectives of this research are to study the Brahmaputra River in Arunachal Pradesh and Assam aiming (1) to assess the fractionation of Cr, Cu, Zn, Pb and Ni in surface sediments and its correlation with turbidity; (2) to study the effect of the chemistry of overlying water on the mobility of metals; (3) to estimate the risk of metal pollution in the sediment of the Brahmaputra River.

## 2. Study Area: Site Description and Geology

The Brahmaputra River is the largest contributor of sand to the ocean in tropical regions [19]. It originates from Kailash Mountain in the Tibetan plateau. The total length of the Brahmaputra River is around 2900 and 916 km of them flows in India. Many tributaries join with the course of the river. The Brahmaputra turns toward the south near Dhubri at the Indo-Bangladesh border and known as Jamuna until it meets the Ganga in Bangladesh. The primary sources of water to the Brahmaputra River are rainfall and meltwater [19]. Most precipitation in the Brahmaputra basin occurs from May to October. The average annual rainfall within the basin is approximately 1500 mm [8].

The entire Brahmaputra drainage system can be classified into five geologically different sub-basins. These are (1) Tibet: the source of the Brahmaputra River; (2) The Eastern Syntaxis (3) the Mishmi Hills; (4) The Himalaya and (5) The Indo-Burmese Ranges. The detailed description of each drainage basin of the Brahmaputra system is explained in [19,22]. The map of the Brahmaputra drainage system is shown in Figure S1 (refer to Supplementary Information (SI)) 

The present study area covers two sub-basins of the Brahmaputra drainage basin: (1) The Eastern Syntaxis and (2) The Himalaya. 

**Figure 1:**
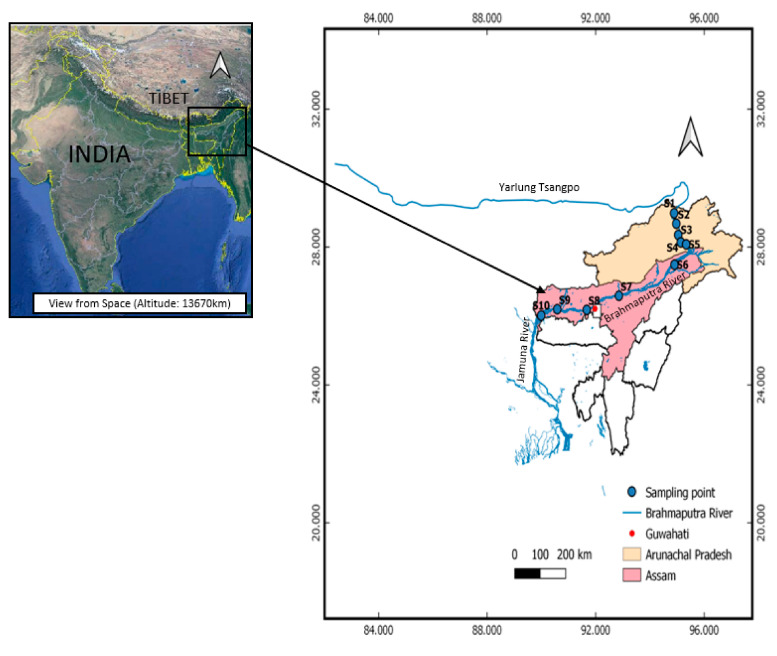
Sampling sites of the Brahmaputra River in Arrunachal Pradesh (S1–S5) and Assam (S6–S10), India (map drawn from Google Earth Pro and using QGIS 3.12 mapping software).

(1) The Eastern Syntaxis: the sampling sites in this region including S1–S5 (Figure 1) are situated the Arunachal Pradesh and the river in this region is known as Siang river. The eastern syntaxis region contains only 4% of the total Brahmaputra area catchment but contributes more than 45% of sediment load [23,24,25]. It consists of highly metamorphized rock formation involving deformed meta-sedimentary, meta-igneous and calc-alkaline plutons surrounded by marbles, phyllites and quartzites [26,27]. 

(2) The Himalaya: the Siang River enters to the southwest and forms a braided river in Assam known as the Brahmaputra River including sampling sites S6–S10. The geology of the Eastern Himalaya comprises higher Himalaya, lesser Himalaya and Sub-Himalayas or Siwaliks [28]. The higher Himalaya consists of schistose quartzite, amphibolite and diopside-bearing banded marble, high grade schists and gneiss rocks intruded by younger tourmaline granite and pegmatite [29]. Other rock units in the higher Himalaya include calc-silicate metamorphic rocks with calcium rich-plagioclase and intercalations of biotitic schists [30]. The lesser Himalayan sequences mainly comprise high-grade metamorphic rocks—i.e., quartzite, granitic gneiss, phyllite, and carbonate. Likewise, Gondwana sequences (latest Carboniferous to lower Cretaceous) made up of 6–7 km thick succession of mainly fluviatile and lacustrine origin predominated by quartzite, slate, shale, sandstone, greywacke, coal beds, arenite, conglomerate and phyllite are also present. The Sub-Himalayas consists of a thick section of Neogene molasses, steep fault scarps and tilted gravel terraces. The sampling sites of this study are shown in Figure 1.

## 3. Materials and Methods

### 3.1. Sampling

Sampling sites S1–S5 lie in Arunachal Pradesh, which is sparsely populated, while the sites S6–S10 are in the Assam state that is a developing state and more populated. Sediments and water samples were collected along the Brahmaputra River in November 2018, and sampling sites are shown in Figure 1. At each sampling site, around 50 g of sediment sample from the surface depth up to 1–5 cm was collected at the shore 20 cm away from the river flow with a metal spoon. Sediment at each site was mixed well and stored in respective polyethylene bags. The collected sediments were transferred to the laboratory and stored in a freezer (−21 °C) until analysis. The overlying water temperature, pH, conductivity, oxidation reduction potential (ORP), dissolved oxygen (DO), turbidity in Nephelometric Turbidity Units (NTU) and total dissolved solids (TDS) were in situ measured by using a micro 800 multiparameter meter, micro 600 DO meter and turbidimeter Plus produced by Palintest, UK. The meters were calibrated before measurement with multiple standard solutions including pH 4.01, pH 7.00 and pH10.01 standards for pH calibration; 84 µS, 1413 µS, 12.88 mS standards for conductivity calibration and 800NTU, 103NTU, 20.7NTU and 1.03NTU standards for turbidity calibration. All the above standard solutions were provided by the manufacturer of the meters (Palintest, UK). The coordinates and elevation of the sampling sites were recorded by using a GPS tracker (Garmin eTrex 10, Lenexa, KS, US). The overlying waters were filtered using 0.45 µm polypropylene syringes and collected in 100 mL pre-rinsed polycarbonate bottle. The particle size distribution of the sediment was determined by sedimentation analysis based on standard ISO 11277:2009 in Eurofins Viljavuuspalvelu Oy, Mikkeli, Finland (ISO, 2009). The grain size distributions are provided in Table S1 (refer to SI). The in situ parameters of the Brahmaputra River are summarized in Table 1. 

### 3.2. Sequential Extraction

In this study, the sequential extraction procedure of metal from the Brahmaputra River bed sediment was followed as proposed by the Community Bureau of Reference (BCR) that is widely applied in many studies [7,11,16,31]. The extraction method is summarized as shown in the flow chart scheme in Figure S2 (refer (SI) Supplementary Information). Residue from the third step was extracted according to the Environmental Protection Agency (EPA) method 3052 (EPA, 1996). The total metal concentration was also determined by using the EPA method 3052—i.e., 0.5 g of samples were digested with 9 mL concentrated HNO_3_ and 3 mL concentrated HF in a microwave (Speedwave two, Berghof, Germany). The temperature was set to increase to 180 ± 5 °C within 10 min and remained heated at 180 ± 5 °C for 9.5 min. 

Sediments were completely dried at 40°C for around 48–60 h in an oven before the BCR procedure. The KS 4000 ic control shaker (IKA, Germany) was used for the agitation of the sediments at room temperature for 16 h. The extraction of supernatant (fractions) from residue in each step were centrifuged at 3000 rpm for 20 min, and the supernatant was transferred into a polyethylene centrifuge tube. The residue was washed with 20 mL deionized water for 15 min in the mechanical shaker and then centrifuged at 3000 rpm for 20 min. The supernatant was decanted, and the residue was subjected to the next steps. 

The extractant was prepared according to the following procedures:

Acetic acid (0.11 M): 25 mL of glacial acetic acid (from Merck, Darmstadt, Germany) was added to about 400 mL of deionized water and made up to the mark of 1000 mL a volumetric flask. Then, 250 mL of the above solution was diluted in 1000 mL to obtain 0.11 M acetic acid.

Hydroxylammonium chloride (0.5 M, pH 1.5): 34.75 g of hydroxylammonium chloride (from Merck, Darmstadt, Germany) was dissolved in deionized water and acidified with concentrated nitric acid to a pH of 1.5 and made up to 1000 mL of deionized water in a volumetric flask.

Hydrogen peroxide (8.8 M): used as the manufacturer (VWR, Belgium) provided. 

Ammonium acetate (1.0 M): 77.08 g of ammonium acetate (from Merck, Darmstadt, Germany) was dissolved in 800 mL of deionized water and acidified to a pH of 2 with concentrated nitric acid and made up to 1000 mL in a volumetric flask.

### 3.3. Analysis

The concentrations of all studied metals in each fraction were measured by Inductively Coupled Plasma-Optical Emission Spectroscopy (ICP-OES) (Agilent 5100, Australia). The method’s accuracy was assessed by the reference solution of the mono-component element (certified reference material (CRM), Sigma Aldrich, St. Louis, MO, USA). Blank and three replicate sample analyses were performed. The blank values were below the detection limit. The detection limits of the metals were Cr, 0.5 μg/L; Cu, 0.5 μg/L; Zn, 0.3 μg/L; Pb, 1.9 μg/L and Ni, 0.9 μg/L. The % Relative Standard Deviation (RSD) of all the analysis of elements was <15%, indicating the high precision of the methods. 

The concentration for total nitrogen, NO_2_ + NO_3_ and NH_4_ of the overlying water was measured using methods based on standard SFS-EN ISO 11905-1:1998 (ISO, 1998), SFS-EN ISO 13395:1997 (ISO, 1998) and SFS 3032:1976 (SFS, 1976), respectively, at the Finnish Environment Institute, Finland. Loss on Ignition (LOI %) was performed to study the sediment’s organic matter content. An empty crucible was ignited at 550 °C for 1 h, cooled and weighed. An amount of 3 g of sediment was transferred to an ignited weighed crucible and heated for 24 h at 105 °C in an oven. Then, the crucible was cooled in a desiccator and weighed and then heated at 550 °C for 4 h in a muffle furnace. The crucible was then cooled and weighted. 

Then,

(1) $$\mathrm{LOI}\;\%=\frac{\mathrm{Sediment}\;\left(\mathrm{heated}\;\mathrm{at}\;105\;{}^{\circ}C\;\mathrm{for}\;24\;h\right)-\mathrm{Sediment}\;\left(\mathrm{ignisted}\;\mathrm{at}\;550\;{}^{\circ}C\;\mathrm{for}\;4\;h\right)}{\mathrm{Sediment}\;\left(\mathrm{heated}\;\mathrm{at}\;105\;{}^{\circ}C\;\mathrm{for}\;24\;h\right)}$$

### 3.4. Quality Assessment and Quality Control

Ultra-pure water was used throughout the dilution procedure, and all reagents were analytical grade unless otherwise stated. Acetic acid (glacial, 100% supra pure, Sigma Aldrich), hydroxylammonium chloride (99.999% trace metal basis, Sigma Aldrich), hydrogen peroxide (30%, stabilized), ammonium acetate (99.999% trace metal basis, Sigma Aldrich) and nitric acid (60%, Suprapur Merck) were used. All glassware was cleaned with nitric acid and rinsed with deionized water before use.

To assess the accuracy of the method, a certified reference material (CRM, BCR 701) was subjected to the BCR protocol. It was observed that the measured values of BCR 701 fractions are in good agreement with their certified value (refer to SI, Table S2). Borah et al. [8] estimated the average total concentration of Cu (40.9 mg/kg) and Zn (51.8 mg/kg) for the Brahmaputra River bed sediment flowing in Assam state that is in good range with the total concentrations obtained in this study as Cu (46.88 mg/kg) and Zn (38.74 mg/kg).

### 3.5. Risk Assessment Code (RAC)

RAC is known as the % of the exchangeable fraction to the total fractions [32,33]. It is widely used to assess risk of mobility and bioavailability. The RAC can be classified into five categories as no risk (≤1%), low risk (1–10%), medium risk (10–30%), high risk (30–50%) and very high risk (>50%). 

## 4. Results 

### 4.1. General Characteristics of Water and Sediment

The overlying water of representative sediments was alkaline with a pH range from 7.4 to 8.1. The dissolved oxygen content of water varied within a narrow range of 8.5 to 12.0 mg/L. A significant variation of turbidity from 73 to 875 NTU (Nephelometric Turbidity Units) was observed. The higher values of turbidity could originate from natural phenomena such as erosion, organic debris and anthropogenic inputs such as industrial effluents, domestic sewage and agriculture waste. The Brahmaputra River had an average total dissolved solid 87.2 mg/L in this study, which is almost identical to the study conducted on same river by Singh et al. [19] having 87.9 mg/L. The TDS value of the Brahmaputra River was lower than its upstream known as the Yarlung Tsangpo from the Tibetan Plateau. The lower TDS concentration of the Brahmaputra River than the Yarlung Tsangpo was likely due to the dilution caused by rainfall and higher runoff. The in situ measurements of the Brahmaputra River are summarized in Table 1. Measured Eh was calculated as measured ORP + 206 − 0.7(t − 25) mV, where t is temperature (°C) [34]. Brahmaputra River sediments’ surface texture was mainly sand (2.0 − 0.06 mm) and then silt (0.06 − 0.002 mm) and least clay (<0.002 mm) The organic matter content (% OM) of the sediment ranged from 0.2 to 1.8%.

### 4.2. Chemical Fractionation 

The sequential extraction procedure allows the estimation of the bioavailability and mobility of metals in the sediment. Metals in sediment can be classified according to BCR procedure as fraction 1 (exchangeable and bound to carbonates), fraction 2 (bound to iron and manganese oxide), fraction 3 (bound to organic matter and sulfides) and fraction 4 (residual fraction).

Fraction 1 (exchangeable and bound to carbonates) is a loosely bound fraction, and metals may readily equilibrate with the aqueous phase. Metals associated with exchangeable fraction vary with the change in the ionic composition of the water, and metal associated with carbonate forms is highly sensitive to pH. In fraction 2, Fe and Mn oxide have an excellent scavenging ability but are sensitive in anoxic conditions [35]. For fraction 3, the peptization properties—i.e., the ability of the organic matter to form a complex with metals through peptization reactions and complexation properties of organic matter such as detritus, living organisms and mineral particles—adhere to metals. However, under the oxidizing environment, the organic matter gets degraded and metal bound to organic matter can be leached. Fraction 4 (residual fraction) involves metals from natural sources attached within the crystal lattice and are not readily bioavailable. 

**Figure 2:**
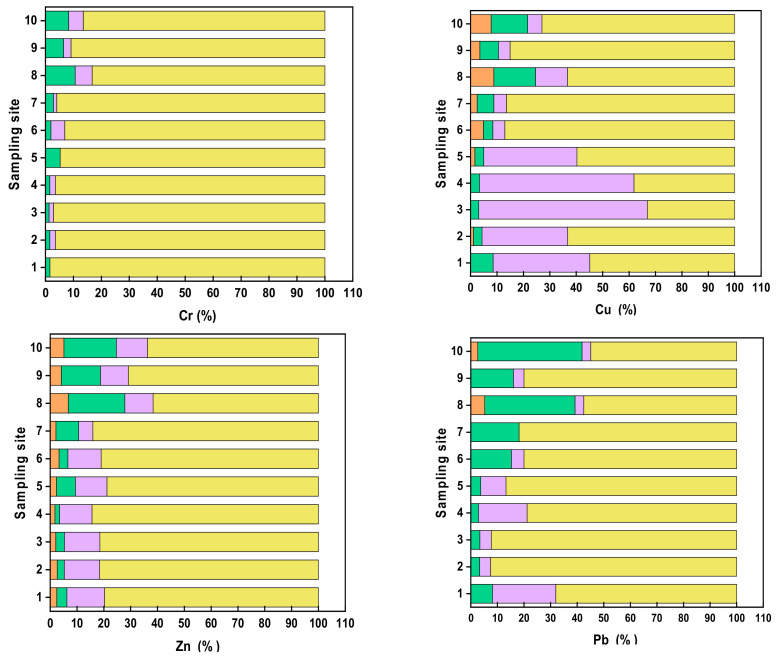

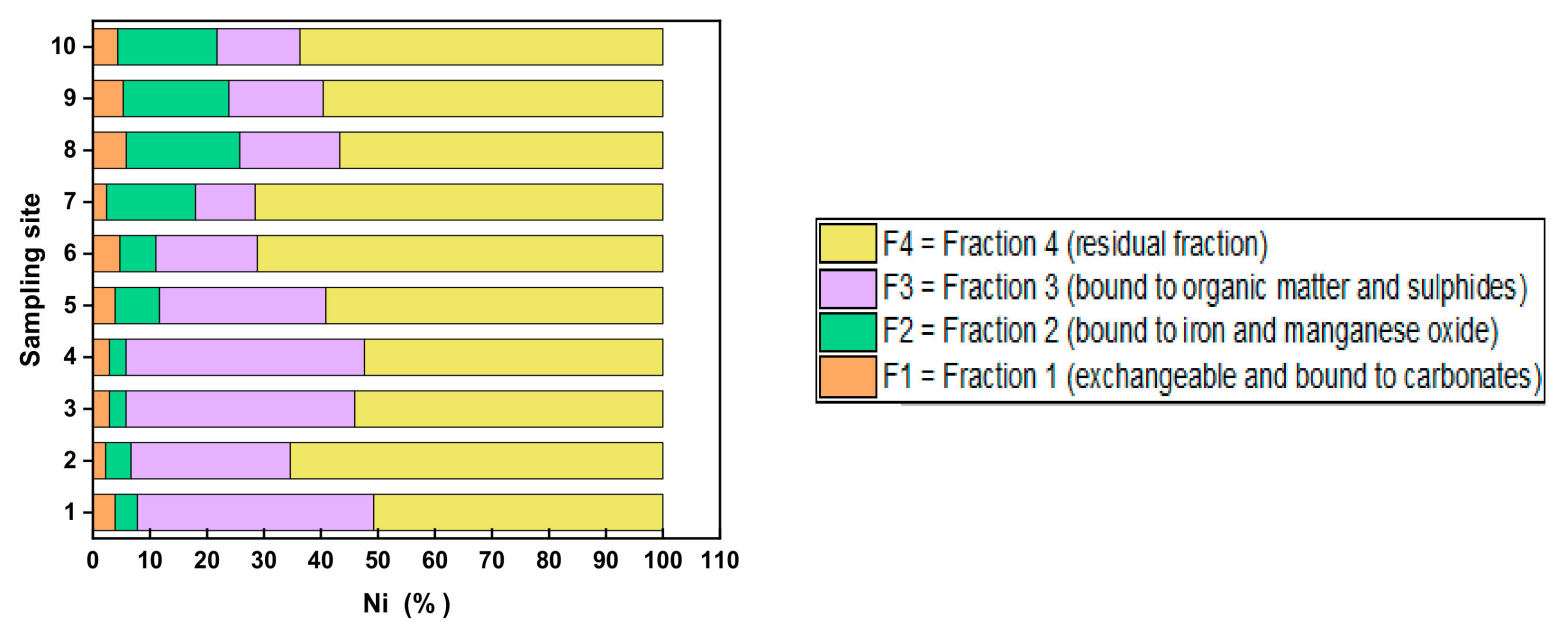
Stacked plot of concentration (%) of different fractions of Cr, Cu, Zn, Pb and Ni.

The fractionation profiles of the metals Cr, Cu, Zn, Pb and Ni (in concentration %) are summarized in Figure 2. 

The fractionation profile of Chromium showed that residual form is a predominant fraction in all the sampling sites, which likely indicates the incorporation of Cr within rocks such as phyllites, amphibolites and/or diopside-bearing banded marbles [26]. Pallavi et al. [17] observed a similar fractionation trend of Cr in the residual fraction. Chromium was also associated with Fe and Mn oxides (F2) with higher concentration at sites S5–S10. This is most likely due to Fe oxyhydroxides enriched in lesser Himalaya and Sub-Himalayas (Siwaliks) rock units. An appreciable amount of Chromium bound to organic matter and sulfide fraction (F3) at S6, S9 and S10 indicated the higher tendency of complexation with organic matter at these sites. Similar findings of Chromium fractionation in river sediments were observed in Mahanadi River system in India, Seyhen River in Turkey and Sungai Buloh in Malaysia [7,11,15]. 

The fractionation profile of Copper indicated that Cu is relatively often bound with fraction 3 at sites S1–S5 and with residual fraction at sites S6-S10. A similar pattern of spatial variation is also reported in Mahanadi River in India by Nemati et al. [7]. In general, Copper is readily influenced by organic complexation due to the strong interaction between the metal and dissolved organic matter [36]. In addition, being strongly chalcophile Cu in mineral form occurs mostly in sulfides. Copper tends to exist with organic and residual fractions, which was also observed in the Yamuna River in India [37]. Copper bound with Iron and Manganese oxides fraction F2 was observed at all sites with the highest portion at S8 and S10. Iron and Manganese oxides can exist as a coating on the particles, as nodules, cement between particles and Cu likely binds the nodules with these oxides. Likewise, the exchangeable fraction was observed at S2 and S5 to S10 with maximum concentration at S8 and S10. The Copper bound with fraction 1 and fraction 2 with the highest concentrations at S8 and S10 indicated an additional anthropogenic source. 

Zinc’s fractionation pattern is Residual fraction > bound to organic matter and sulfide fraction > Fe and Mn oxide bound fraction > Exchangeable fraction. However, an additional concentration of Zinc bound to Iron and Manganese oxides fraction than the oxidized fraction (bound to organic matter and sulfides) was observed at sites from S7 to S10. Complexing of Zinc with organic matter is important only where the concentration of organic carbon is relatively high [37]. 

The chemical fraction of Lead in the Brahmaputra River is predominantly residual. However, a major variation in a trend of fractions of Pb from S1 to S10 was observed. The exchangeable fractions were observed in S8 and S10, which indicates human-caused pollution. Lead existed in fraction 2 at all sites but relatively abundant at S6–S10, and Lead also existed in fraction 3 relatively abundant at site S1-S5. A similar observation of Lead in fraction 2 and residual fraction was found in a previous study [17]. 

Nickel showed an almost similar fractionation profile as Zinc indicating the common sources of fractions of the Nickel and Zinc in the Brahmaputra River. However, exchangeable fraction in Nickel was generally more abundant than in Zinc. The dominant fraction of Nickel from S1 to S5 was residual and bound to carbonates. Nickel in fraction 3 decreased from S6 to S10 with an increase in Ni bound to Fe-Mn oxide fraction (F2) and exchangeable fraction. The exchangeable fraction of Nickel existed at all sites.

## 5. Discussion

### 5.1. The Interrelationship between the Overlying Water and Metal Species in the Sediment

The interrelationship between the overlying water condition and different fractions of surface sediments can be studied by using multivariate statistical analysis. In this study, the principal component analysis and cluster analysis were applied using IBM SPSS statistical software.

#### 5.1.1. Principal Component Analysis

**Figure 3:**
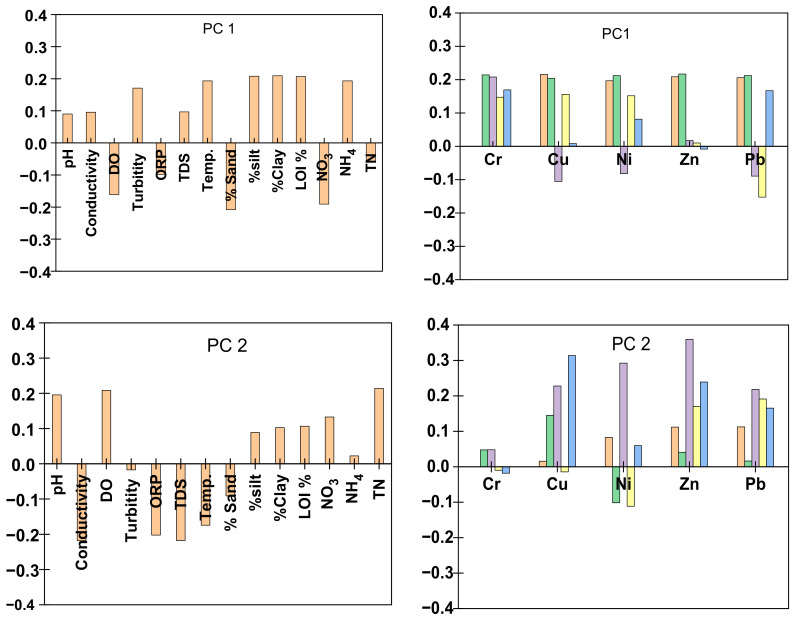

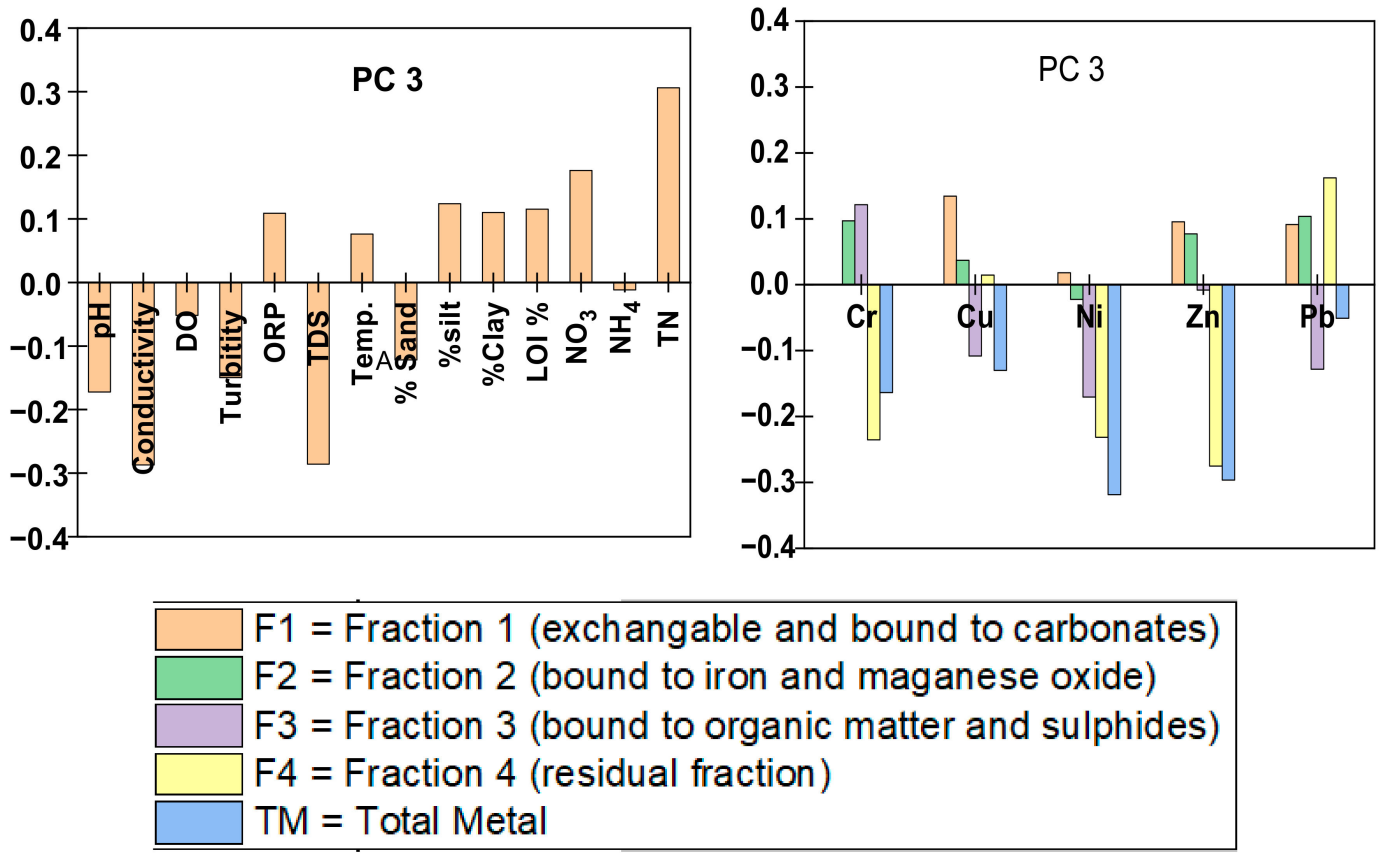
Sub-plots of variable loadings on principal component 1 (PC 1), principal component 2 (PC 2), principal component 3 (PC 3).

Principal component analysis (PCA) was executed on total concentrations and four fractions of Cu, Ni, Zn, Cr, Pb and overlying water parameters, which are turbidity, pH, conductivity, dissolved oxygen (DO), oxidation reduction potential (ORP), total dissolved solids (TDS), temperature, surface texture of the sediment, loss of ignition (%) of sediment and nitrogen contents in the water. The first three components of the PCA captured 79% of the variation, which were extracted, and sub-plotted as shown in Figure 3. Data were scaled to unit variance and centered for PCA.

Component 1 explains 48% of the variance, and it is highly contributed to by the fraction 1 (Cr, Zn, Ni, Cu, Pb), fraction 2 (Zn, Cu, Cr, Ni, Pb), surface texture (0.06–0.002 mm for silt % and <0.002 mm for clay%), % LOI (organic matter), turbidity, NH_4_ and temperature. Fraction 1 of BCR sequential extraction procedures indicated the most labile and bioavailable that could be from anthropogenic sources. The turbidity was highly correlated with fractions 1 and 2 of all metal with correlation coefficient value r > 0.50 (refer to SI, Table S3). Likewise, the temperature was positively correlated with the exchangeable fraction of the metals. This indicates a possible direct relation of bioavailability and toxicity of metals with the temperature and turbidity of the overlying water. PC scores revealed an increasing trend in sampling sites towards downstream (particularly sites S8 and S10; refer to SI, Figure S3).

The ammonium concentration in the Brahmaputra River varied from its flow in Arunachal Pradesh (S1–S5) and the Assam (S6–S10). NH_4_ was predominant at Assam, particularly at S7 and S8, which had the maximum NH_4_ values (refer to SI, Table S4) that might be attributed to anthropogenic inputs. The higher concentration of NH_4_ in the overlying water at these sites has a potential risk due to the strong correlation between NH_4_ and metals in the exchangeable fractions (refer to SI, Table S3). NH_4_ can replace the metals in the exchangeable fraction, and subsequently, the metals can release into the overlying water [15,37]. The presence of a higher concentration of the exchangeable fraction at site S8 indicates that this site is polluted from a direct point source which could be from industrial effluent or domestic waste. The average values of NO_3_ at sites S1–S5 and S6–S10 were 278 and 120 µg/L, respectively. 

Component 2 contributes 18% of the variance. Fraction 3 and DO are the major parameters involved. In fraction 4, Cu and Zn were also observed. The results showed that DO in the overlying water had a significant positive correlation with fraction 3, which indicates that any resuspension of the bottom sediments has a potential risk because the metals bound to organic matter, and sulfide can be oxidized and released to the overlying water [38,39,40]. The uncertainties of heavy floods events in the Brahmaputra basin with the recent increase in the amplitude of rainfall fluctuation could further maximize the potential risk associated with resuspension of the sediment and leaching of metals into overlying water [41,42]. Studies reported that DO of the overlying water plays a vital role in the content of metals that are bound with sulfides and organic matter [5,38,43,44]. Under aerobic conditions, the decomposition of the organic matter was reinforced due to the presence of oxygen, and metals were released. Similarly, sulfides were oxidized to sulfates and metals were released from the metal sulfide. Most sites had a positive PC score on PC2, but sites S7 and S9 loaded negatively due to different pollution profile at these two sites (refer to SI, Figure S3).

Component 3 contributed 13% of the variance. Major variables involved in Component 3 were TDS, conductivity, total nitrogen, fraction 4 (Ni, Cr, Zn) and total concentration (Ni, Zn). The residual fraction of Pb could be from a source other than Cr and Ni, as they were negatively correlated (refer to SI, Table S3). The residual fraction was embedded within the lattice of the bedrock of the sediment. Thus, component 3 can be considered as a natural factor. This indicates that metals are associated with breakdown (weathering) of the bedrock, and during the weathering process, the major ions are dissolved to build up the TDS of the Brahmaputra River. Likewise, a positive correlation between Cr and Ni with TDS was observed.

#### 5.1.2. Cluster Analysis

**Figure 4:**
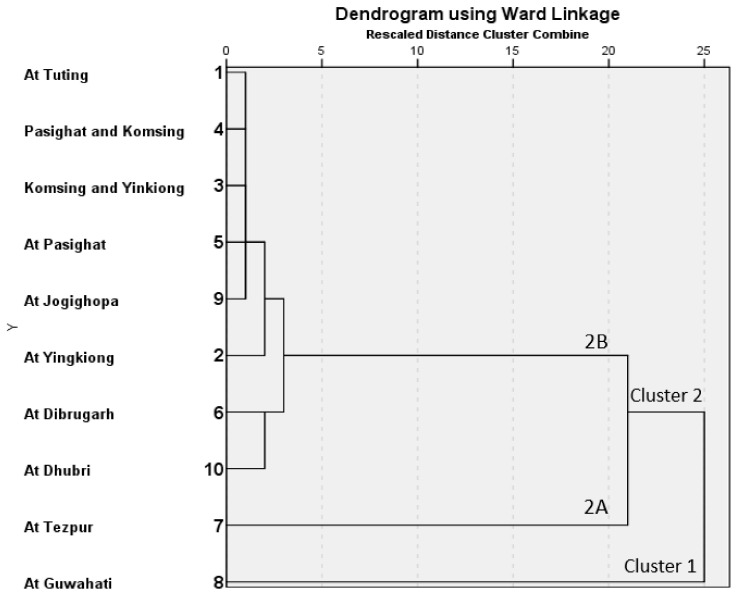
Cluster analysis dendrogram of the sampling sites.

All parameters that were used in PCA were subjected to Hierarchical cluster analysis to build clusters with similar characteristics of the sampling sites. Ward’s method using squared Euclidean distances as measures of similarity was implemented as it uses more information on cluster contents than other methods. It has a small distorting effect and proved to be a useful grouping mechanism. The sampling sites can be classified into three major clusters (Figure 4). Based on cluster analysis, site 8 was classified as cluster 1, and all other sites were included in cluster 2 (Figure 4). Site 8 was distinguished as the most polluted site. According to metal fractionation and the chemistry of the overlying water, site 8 showed the highest concentrations of exchangeable fractions, NH_4_ and turbidity (Figure 4 and refer to SI, Table S4).

Moreover, site 8 showed the highest value of the risk assessment code (refer to SI, Table S5). Site 8 is located at Guwahati, which is the largest city in the Indian state of Assam and the largest urban area in Northeast India. This indicates that site 8 receives considerable amounts of anthropogenic inputs. Although the remaining sites were included in cluster 2, site 7 was distinguished from other sites as a sub-cluster 2A. Site 7 was ordered as the second site and included considerable concentrations of turbidity and NH_4_. In the sub-cluster 2B, the sites 6 and 10 were linked together and formed a distinctive cluster. It was found that these sites were characterized by noticeable concentrations of exchangeable metals. Sites 6 and 10 are located across urban regions. The population (density/km^2^) at each sampling site is shown Table S4 (refer to SI). The dendrogram of current studies provides useful information on the contribution of the anthropogenic inputs to the metal pollution and the vicinity sampling sites.

### 5.2. Risk Assessment Code (RAC) and Environmental Implications for the Metal Fractionation

The range of RAC values was 1.8–6.8% for Zn; 2.2–5.9% for Ni; up to 8.8% for Cu and up to 5.2% for Pb. The RAC value of Cu, Ni, Zn and Pb at each sampling site is summarized in Table S5 (refer to SI). Zn and Ni were in the low risk category at all sampling sites. The RAC value of Cu was in the low risk category with major values ranging below 3.5% except at sites S6 (4.9%), S8 (8.8%) and S10 (7.8%). Pb had no risk at all in the sampling sites except S8 and S10 having >1%, which implies point sources of pollution at respective sampling sites. 

**Figure 5:**
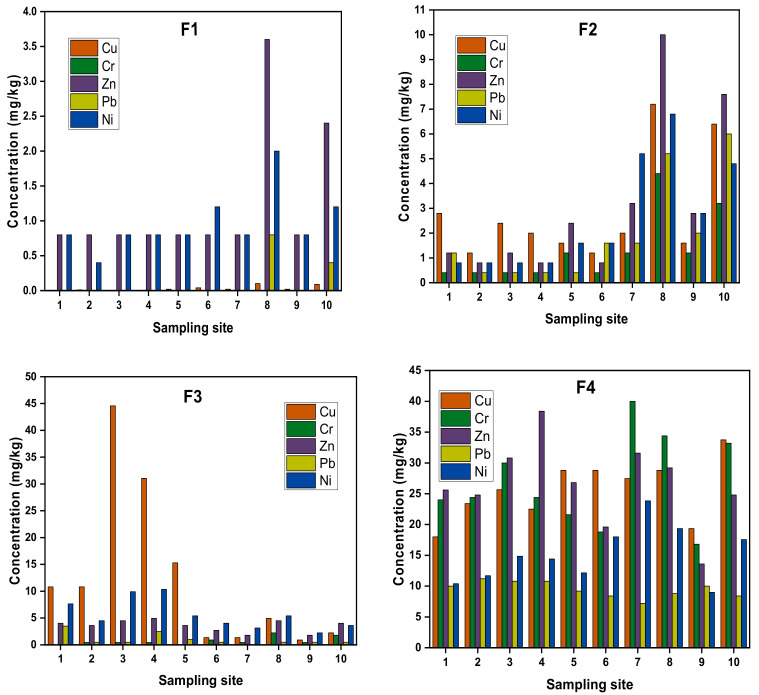
Plot of sampling site(s) vs concentration (mg/kg).

The metals (Cr, Cu, Ni, Zn, Pb) in the four different fractions are summarized in Figure 5. 

Fraction 1 (exchangeable and bound to carbonate) represented the most bioavailable fraction and can readily leach into the overlying water. The largest city in Assam is located at S8 and the highest concentrations of all studied metals in fraction 1 were recorded at S8 (see Figure 5 and refer to SI, Table S5). A considerable concentration of the exchangeable Pb was observed at S10 (2.61%), which is 50–80 m away from a ship port from India to Bangladesh and could have contributed, along with municipal waste and industrial effluents, to the river. The Brahmaputra River is a lifeline for agriculture and domestic use for all the locality along its course. Cr was not detected in fraction 1. 

Fraction 2 (bound to Fe and Mn oxide) of metals Cu, Cr, Zn, Pb and Ni was observed in all the fractions with appreciable amounts. The highest concentrations of metals were observed at S8. The observation of the increasing trend of fraction 1 and fraction 2 at S6 to S10 in comparison to S1 to S5 shows the increase in pollutants. This observation agreed well with the fact that the sites S6–S10 are located along with the highest population sites of Northeast India.

All the studied metals were detected in fraction 3 (bound to organic matters and sulfides) (Figure 5). Although the highest percentages of organic matter were found at S8 and S10 (refer to SI, Table S4), the maximum concentration of fraction 3 of Cu was recorded at S3 and S4. Organic ligands tend to stabilize metals via the formation of complexes. However, this process can take several hours [45,46]. Accordingly, if quick exchanges occur in the sediments, in this case, the organic matter may play a limited role in metal fixation, and this phenomenon could have applied at sites S8 and S10 [46]. In addition, Ciazela et al. [47] observed that the highest rate of metal extraction in the bottom sediment of Oxbow lakes by using ethylenediaminetetraacetic acid (EDTA extracts portions of metals that are mostly associate with organic matter) was achieved for the medium grain size fraction (0.25 to 1 mm of particle diameter). Similarly, in this study, S8 and S10 had a higher % of the lower grain size (<0.002 mm) than the remaining sites, which might have led to the lower concentration of fraction 3. So, it can be concluded that grain size is also an important factor of determining the metal concentration in the sediments. Additionally, Luther et al. [48] stated that the sulphidization of organic matter (e.g., thiol and polysulfide functional groups) can result in an underestimation of the binding capacity of organic matter.

Fraction 4 (Residual fraction) had the highest concentration of all the metals of the current study sites, which implies that the natural factor highly dominates the chemistry of the Brahmaputra River. However, the increase in the concentration of fractions 1 and 2 at S6–S10 shows the risk of water pollution and the need to take countermeasures for the industrial sewage dumping and throwing of domestic waste into water [49,50]. Thus, overall observations of the chemical forms of metals and the characteristics of overlying water of the Brahmaputra River highlight the importance of the continuous monitoring of the river quality and environmental management practices. The spatiotemporal dynamics of metal in Brahmaputra River’s water observed multi-metal co-contamination in different groups, which indicated various contributing factors including lithological sources, anthropogenic, climatic and terrigenous sources to the metal content in the river [12].

## 6. Conclusions

The BCR protocol was applied for the fractionation of Cr, Ni, Zn, Pb and Cu in the Brahmaputra River surface sediments. Principal component analysis and risk assessment code evaluated the relationship between the overlying water’s chemistry, the fractions of metals and the extent of metal pollution in the river surface sediment. Cluster analysis classified the sites based on the level of contamination, and the findings showed that site 8 had the highest concentration of fraction 1 and fraction 2. The dominance of residual fraction in all metal implies that the geochemical composition of the area largely influences the Brahmaputra River surface sediment. However, sites S8, S10 and S7, which are at the largest state of the northeast and nearest vicinity to industries are the most contaminated. The exchangeable fraction of Zn and Ni was observed at all sites, while Pb and Cu were found in some downstream sites, indicating the point source of anthropogenic inputs. The turbidity, NH_4_ and temperature of the overlying water are highly correlated with the metals’ exchangeable fraction. The risk assessment code (RAC) of the studied metal indicated that the river’s current state is at low risk. However, the significant fluctuation of the turbidity of the Brahmaputra River poses a potential risk of pollution, and its association with the most labile fraction of metal demonstrated the importance of continuous assessment of the water quality of the river. The dissolved oxygen concentration of the overlying water is significantly correlated with the oxidizable fraction, which implies that any resuspension of the bottom sediments may lead to the release of metals bound to organic matter and/or sulfide into the overlying water during the oxidation process. These findings of this study could support the development of practical management strategies, creating awareness of the prevention of metal pollution, and future monitoring research on the river metal pollution.

## Figures and Tables

**Table 1:** Sampling site details and parameters of the Brahmaputra River.

Sediment No.	Date	Latitude	Longitude	Elevation (m)	pH	Conduc- Tivity (uS)	DO (mg/L)	Turbidity (NTU)	Measured Eh (mV)	TDS (ppm)	Temp. (°C)
S1	13/11/2018	28.98097	94.89255	414	7.98	383.8	11.29	207	135.78	90.84	12.6
S2	12/11/2018	28.677	94.97366	287	7.52	278.5	10.52	72.9	146.81	65.60	15.7
S3	11/11/2018	28.35093	95.04578	215	7.93	353.2	12.00	258	135.76	82.60	14.2
S4	11/11/2018	28.13514	95.14008	168	7.78	349.8	11.54	179	143.33	82.80	14.1
S5	10/11/2018	28.07835	95.33553	132	7.97	312.6	11.50	207	135.70	73.93	15.0
S6	9/11/2018	27.48809	94.90676	83.0	8.08	346.5	8.490	361	120.40	82.10	22.0
S7	20/11/2018	26.58656	92.85958	57.0	7.73	897.1	9.050	557	141.31	211.0	22.7
S8	21/11/2018	26.18033	91.67194	23.0	8.04	439.1	8.820	875	120.19	104.1	24.3
S9	22/11/2018	26.1983	90.58509	27.0	7.39	381.7	10.04	134	160.72	90.34	20.4
S10	22/11/2018	26.01339	89.99179	15.0	7.92	473.9	9.260	194	125.84	112.4	25.8

## Supplementary Materials

The following are available online at https://www.mdpi.com/1660-4601/17/24/9214/s1, Figure S1: The Brahmaputra drainage system (Map modified after Singh et al. [19]). Figure S2: Flow chart of the sequential extraction procedure. Figure S3: PC Scores plots of Principal Component at respective sampling sites. Table S1: Grain size distribution (in %) of the sediment sampling sites. Table S2: Certified and measured values of fractions of certified reference sediment BCR-701. Table S3: Correlation table of the overlying water parameter and chemical forms of the surface sediment of the Brahmaputra River sediment. Table S4: Nitrogen contents of the overlying water, LOI% of the sediments and population (density/km^2^) of the studied sampling sites. Table S5: RAC values of Cu, Ni, Zn and Pb at the difference sampling sites no detection of Cr in the exchangeable fraction at all sites.

## References

[1] Frémion F., Bordas F., Mourier B., Lenain J.F., Kestens T., Courtin-Nomade A. (2016). Influence of dams on sediment continuity: A study case of a natural metallic contamination. Sci. Total Environ..

[2] Chen M., Ding S., Chen X., Sun Q., Fan X., Lin J., Ren M., Yang L., Zhang C. (2018). Mechanisms driving phosphorus release during algal blooms based on hourly changes in iron and phosphorus concentrations in sediments. Water Res..

[3] Artifon V., Zanardi-Lamardo E., Fillmann G. (2019). Aquatic organic matter: Classification and interaction with organic microcontaminants. Sci. Total Environ..

[4] Giuliano V., Pagnanelli F., Bornoroni L., Toro L., Abbruzzese C. (2007). Toxic elements at a disused mine district: Particle size distribution and total concentration in stream sediments and mine tailings. J. Hazard. Mater..

[5] Kang M., Tian Y., Peng S., Wang M. (2019). Effect of dissolved oxygen and nutrient levels on heavy metal contents and fractions in river surface sediments. Sci. Total Environ..

[6] Du Laing G., Rinklebe J., Vandecasteele B., Meers E., Tack F. (2008). Trace metal behaviour in estuarine and riverine floodplain soils and sediments: A review. Sci. Total Environ..

[7] Nemati K., Kartini N., Bakar A., Abas M.R., Sobhanzadeh E. (2011). Speciation of heavy metals by modified BCR sequential extraction procedure in different depths of sediments from Sungai Buloh, Selangor, Malaysia. J. Hazard. Mater..

[8] Borah R., Taki K., Gogoi A., Das P., Kumar M. (2018). Contemporary distribution and impending mobility of arsenic, copper and zinc in a tropical (Brahmaputra) river bed sediments, Assam, India. Ecotoxicol. Environ. Saf..

[9] Vosoogh A., Saeedi M., Lak R. (2017). Metal fractionation and pollution risk assessment of different sediment sizes in three major southwestern rivers of Caspian Sea. Environ. Earth Sci..

[10] Vosoogh A., Saeedi M., Lak R. (2016). Heavy metals relationship with water and size-fractionated sediments in rivers using canonical correlation analysis (CCA) case study, rivers of south western Caspian Sea. Environ. Monit. Assess..

[11] Davutluoglu O.I., Seckin G., Ersu C.B., Yilmaz T., Sari B. (2011). Heavy metal content and distribution in surface sediments of the Seyhan River, Turkey. J. Environ. Manag..

[12] Gogoi A., Taki K., Kumar M. (2020). Seasonal dynamics of metal phase distributions in the perennial tropical (Brahmaputra) river: Environmental fate and transport perspective. Environ. Res..

[13] Hu L. (2014). Effects of sediment geochemical properties on heavy metal bioavailability. Environ. Int..

[14] Liu Q., Jia Z., Li S., Hu J. (2019). Assessment of heavy metal pollution, distribution and quantitative source apportionment in surface sediments along a partially mixed estuary (Modaomen, China). Chemosphere.

[15] Sundaray S.K., Nayak B.B., Lin S., Bhatta D. (2011). Geochemical speciation and risk assessment of heavy metals in the river estuarine sediments-A case study: Mahanadi basin, India. J. Hazard. Mater..

[16] Pueyo M., Mateu J., Rigol A., Vidal M., López-Sánchez J.F., Rauret G. (2008). Use of the modified BCR three-step sequential extraction procedure for the study of trace element dynamics in contaminated soils. Environ. Pollut..

[17] Pallavi D., Kumar M., Sarma K.P. (2015). Speciation of Heavy Metals in Surface Sediment of the Brahmaputra River, Assam, India. J. Environ. Res. Dev..

[18] Berner E.K., Berner R.A. (1996). Global Environment: Water, Air and Geochemical Cycles.

[19] Singh S.K., Sarin M.M., France-Lanord C. (2005). Chemical erosion in the eastern Himalaya: Major ion composition of the Brahmaputra and δ13C of dissolved inorganic carbon. Geochim. Cosmochim. Acta.

[20] PTI (2017). Causes behind Brahmaputra Turning Black Could Be Natural: Union Minister. https://economictimes.indiatimes.com/news/politics-and-nation/causes-behind-brahmaputra-turning-black-could-be-natural-union-minister/articleshow/61911643.cms.

[21] PTI (2017). Brahmaputra River In Assam Has Changed Its Colour and Has Turned Muddy. https://swachhindia.ndtv.com/brahmaputra-river-assam-changed-colour-turned-muddy-15567/.

[22] Verma S., Mukherjee A., Mahanta C., Choudhury R., Mitra K. (2016). Influence of geology on groundwater–sediment interactions in arsenic enriched tectono-morphic aquifers of the Himalayan Brahmaputra river basin. J. Hydrol..

[23] Garzanti E., Vezzoli G., Ando S., France-lanord C., Singh S.K., Foster G. (2004). Sand petrology and focused erosion in collision orogens: The Brahmaputra case. Earth Planet. Sci. Lett..

[24] Goodbred S.L., Paolo P.M., Ullah M.S., Pate R.D., Khan S.R., Kuehl S.A., Singh S.K., Rahaman W. (2014). Piecing together the Ganges-Brahmaputra-Meghna river delta: Use of sediment provenance to reconstruct the history and interaction of multiple fluvial systems during Holocene delta evolution. Bull. Geol. Soc. Am..

[25] Singh S.K., France-Lanord C. (2002). Tracing the distribution of erosion in the Brahmaputra watershed from isotopic compositions of stream sediments. Earth Planet. Sci. Lett..

[26] Burg J.-P., Oberli F., Maurin J.-C., Davy P., Burg J.-P., Diao Z., Meier M., Seward D., Nievergelt P. (1998). The Namche Barwa syntaxis: Evidence for exhumation related to compressional crustal folding. J. Asian Earth Sci..

[27] Verma S., Mukherjee A., Mahanta C., Choudhury R. (2019). Applied Geochemistry Arsenic fate in the Brahmaputra river basin aquifers: Controls of geogenic processes, provenance and water-rock interactions. Appl. Geochem..

[28] Gansser A. (1964). Geology of the Himalayas.

[29] Dutta S.K., Gill G.K.S., Srinivasan J. (1983). Geology of the Subansiri and Kamala Valleys.

[30] Tipper E.T., Bickle M.J., Galy A., West A.J., Pomiès C., Chapman H.J. (2006). The short term climatic sensitivity of carbonate and silicate weathering fluxes: Insight from seasonal variations in river chemistry. Geochim. Cosmochim. Acta.

[31] Zimmerman A.J., Weindorf D.C. (2010). Heavy Metal and Trace Metal Analysis in Soil by Sequential Extraction: A Review of Procedures. Int. J. Anal. Chem..

[32] Ke X., Gui S., Huang H., Zhang H., Wang C., Guo W. (2017). Ecological risk assessment and source identification for heavy metals in surface sediment from the Liaohe River protected area, China. Chemosphere.

[33] Yang J., Chen L., Liu L.Z., Shi W.L., Meng X.Z. (2014). Comprehensive risk assessment of heavy metals in lake sediment from public parks in Shanghai. Ecotoxicol. Environ. Saf..

[34] Ioka S., Muraoka H., Suzuki Y. (2017). Redox potential of shallow groundwater by 1-month continuous in situ potentiometric measurements. Appl. Water Sci..

[35] Tessier A., Campbell P.G.C., Bisson M. (1979). Sequential Extraction Procedure for the Speciation of Particulate Trace Metals. Anal. Chem..

[36] Drever J.I. (1997). The Geochemistry of Natural Waters Surface and Ground Water Environments.

[37] Jain C.K. (2004). Metal fractionation study on bed sediments of River Yamuna, India. Water Res..

[38] Atkinson C.A., Jolley D.F., Simpson S.L. (2007). Effect of overlying water pH, dissolved oxygen, salinity and sediment disturbances on metal release and sequestration from metal contaminated marine sediments. Chemosphere.

[39] Simpson S.L., Apte S.C., Batley G.E. (1998). Effect of short-term resuspension events on trace metal speciation in polluted anoxic sediments. Environ. Sci. Technol..

[40] Vidal-Durà A., Burke I.T., Stewart D.I., Mortimer R.J.G. (2018). Reoxidation of estuarine sediments during simulated resuspension events: Effects on nutrient and trace metal mobilisation. Estuar. Coast. Shelf Sci..

[41] Deka R.L., Mahanta C., Pathak H., Nath K.K., Das S. (2013). Trends and fluctuations of rainfall regime in the Brahmaputra and Barak basins of Assam, India. Theor. Appl. Climatol..

[42] Kalnejais L.H., Martin W.R., Signell R.P., Bothner M.H. (2007). Role of sediment resuspension in the remobilization of particulate-phase metals from coastal sediments. Environ. Sci. Technol..

[43] Fan B., Zhao Z., Tao F., Liu B., Tao Z., Gao S. (2014). Characteristics of carbonate, evaporite and silicate weathering in Huanghe River basin: A comparison among the upstream, midstream and downstream. J. Asian Earth Sci..

[44] Zheng S., Wang P., Wang C., Hou J., Qian J. (2013). Distribution of metals in water and suspended particulate matter during the resuspension processes in Taihu Lake sediment, China. Quat. Int..

[45] Louis Y., Garnier C., Lenoble V., Mounier S., Cukrov N., Omanović D., Pižeta I. (2009). Kinetic and equilibrium studies of copper-dissolved organic matter complexation in water column of the stratified Krka River estuary (Croatia). Mar. Chem..

[46] Charriau A., Lesven L., Gao Y., Leermakers M., Baeyens W., Ouddane B., Billon G. (2011). Trace metal behaviour in riverine sediments: Role of organic matter and sulfides. Appl. Geochem..

[47] Ciazela J., Siepak M., Wojtowicz P. (2018). Tracking heavy metal contamination in a complex river-oxbow lake system: Middle Odra Valley, Germany/Poland. Sci. Total Environ..

[48] Luther G.W., Church T.M. (1992). An overview of the environmental chemistry of sulphur in wetland systems. Sulphur Cycling on the Continents.

[49] Paul A., Deka J., Gujre N., Rangan L., Mitra S. (2019). Does nature of livelihood regulate the urban community’s vulnerability to climate change? Guwahati city, a case study from North East India. J. Environ. Manag..

[50] Sarmah T., Das S. (2018). Urban flood mitigation planning for Guwahati: A case of Bharalu basin. J. Environ. Manag..

