# Supplementary material for: Metal Fractionation in Surface Sediments of the Brahmaputra River and Implications for Their Mobilization
Source: Int J Environ Res Public Health. 2020 Dec 9;17(24):9214. doi: 10.3390/ijerph17249214 (PMC7764534; doi:10.3390/ijerph17249214)
Supplement: Supplementary file 1 [file ijerph-17-09214-s001.pdf]

International Journal of
Environmental Research and Public Health

MDPI

Metal fractionation in surface sediments of the Brahmaputra River and implications for their mobilization

Supplementary Information (SI)

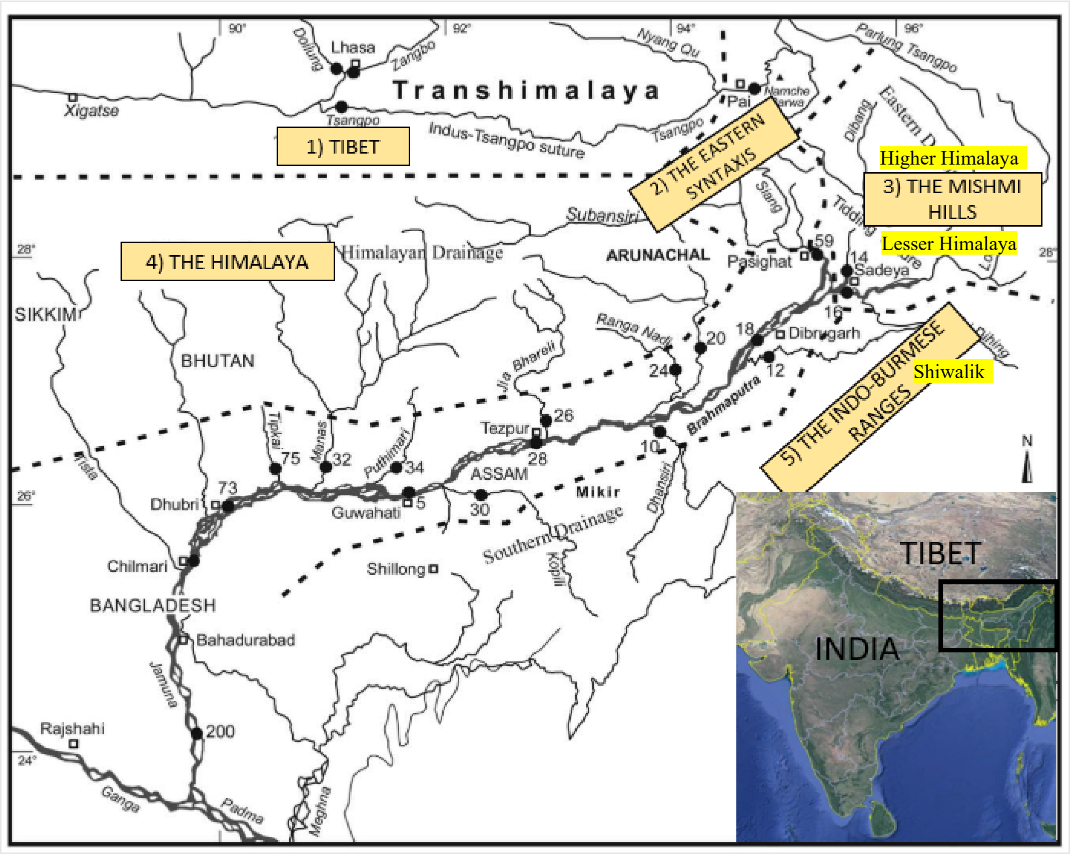

Figure S1. The Brahmaputra drainage system including 1) Tibet 2) The Eastern Syntaxis 3) The Mishmi Hills 4) The Himalaya 5) The Indo-Burmese Ranges (Map modified after Singh et al. [19])

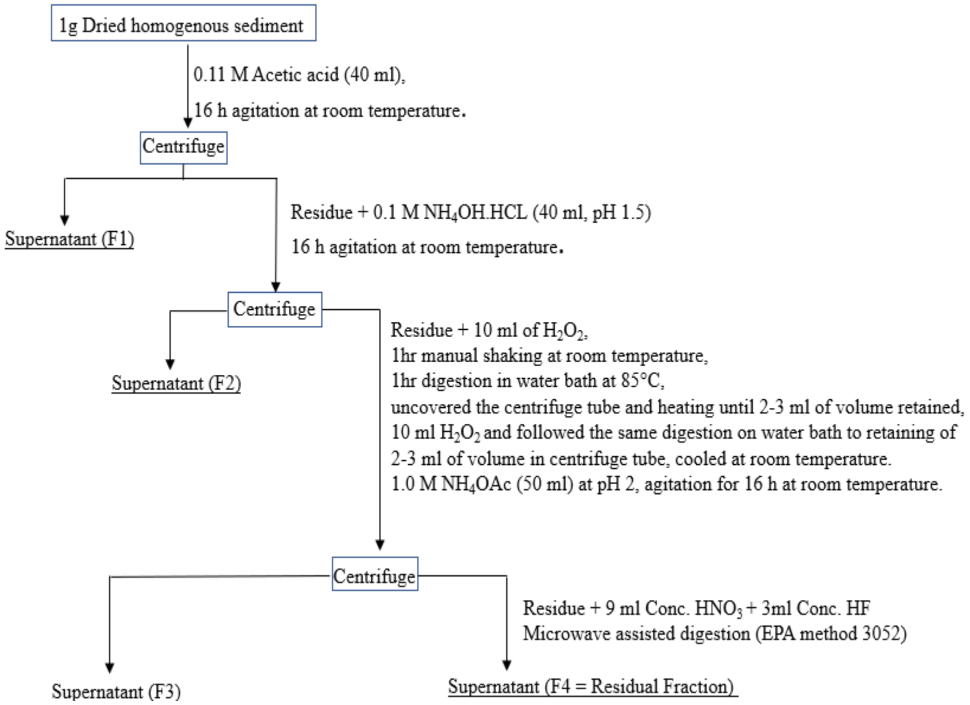

Figure S2. Flow chart of the sequential extraction procedure

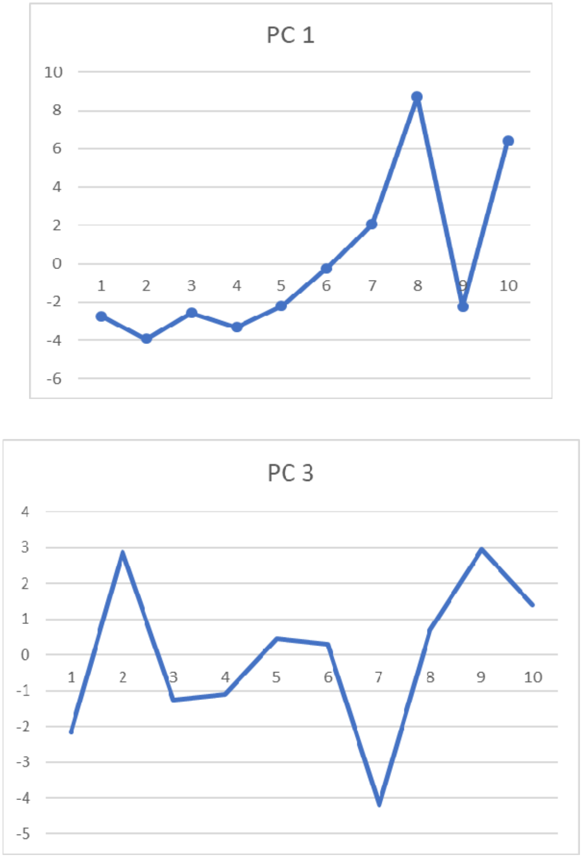

Figure S3. *PC Scores* plot of Principal Component at respective sampling sites

Table S1 Grain size distributions (in %) of the sediment sampling sites

Site no.	Sand%	Silt%	Clay%
S1	99	0	1
S2	99	0	1
S3	99	0	1
S4	99	0	1
S5	99	0	1
S6	98	1	1
S7	99	0	1
S8	80	16	4
S9	98	1	1
S10	78	18	4

The classification of the grain size range is Clay(<0.002mm); Silt (0.06-0.002mm); Sand (2.0-0.06mm); Gravel (20.0-2.0); Cobble (>20mm). The sediment grain size in this study comes under clay, silt and sand classification.

Table S2. Certified and measured values of fractions of Certified reference sediment BCR -701

Steps	Cr	Cu	Ni	Zn	Pb
F1[1]					
Certified	2.3 ± 0.2	49.3 ± 1.7	15.4 ± 0.9	205± 6	3.2 ± 0.2
Measured	2.0± 0.1	42.4 ± 0.8	14.8 ± 1.2	200 ± 13	3.2 ± 0.4
F2[2]					
Certified	45.7 ± 2.0	124 ± 3	26.6 ± 1.3	114 ± 5	126.0 ± 3.0
Measured	49.0 ± 0.2	122 ± 7	28.8 ± 1.6	110 ± 1	130.0 ± 1.7
F3[3]					
Certified	143 ± 7	55 ± 4	15.3 ± 0.9	46 ± 4	9.3 ± 2.0
Measured	120 ± 5	45 ± 2	11.7 ± 0.5	34 ± 2	6.5 ± 0.1

[1]Fraction 1 (exchangeable and bound to carbonates); [2]Fraction 2 (bound to iron and manganese oxide); [3]Fraction 3 (bound to organic matter and sulphides)

Table S3. Correlation table of the overlying water parameter and chemical forms of the surface sediment of the Brahmaputra River Sediment

	pH	Conduc-tivity	DO (mg/L)	Turbi-dity (NTU)	ORP (mV)	TDS (ppm)	Temp.	Cr F2	Cr F3	Cr F4	Cr TM	Cu F1	Cu F2	Cu F3	Cu F4	Cu TM	Ni F1	N F2	Ni F3	Ni F4	Ni TM	Zn F1	Zn F2	Zn F3	Zn F4	Zn TM	Pb F1	Pb F2	Pb F3	Pb F4	Pb TM	% Sand	% silt	% Clay	% LOI	NO_3 (ug/L)	NH_4 (ug/L)	TN (ug/L)
**pH**	1.00																																				
**Conductivity**	-0.06	1.00																																			
**DO (mg/L)**	-0.10	-0.45	1.00																																		
**Turbidity**	0.44	0.49	-0.57	1.00																																	
**ORP (mV)**	-0.96	0.14	0.18	-0.44	1.00																																
**TDS (ppm)**	-0.05	1.00	-0.46	0.49	0.13	1.00																															
**Temp.**	0.08	0.49	-0.91	0.54	-0.12	0.49	1.00																														
**Cr F2**	0.26	0.22	-0.53	0.65	-0.29	0.22	0.73	1.00																													
**Cr F3**	0.31	0.11	-0.67	0.62	-0.42	0.12	0.79	0.88	1.00																												
**Cr F4**	0.22	0.74	-0.28	0.60	-0.24	0.74	0.42	0.51	0.45	1.00																											
**Cr TM**	0.19	0.66	-0.38	0.56	-0.10	0.67	0.54	0.68	0.49	0.70	1.00																										
**Cu F1**	0.36	0.15	-0.71	0.59	-0.43	0.15	0.84	0.93	0.94	0.40	0.54	1.00																									
**Cu F2**	0.40	0.15	-0.38	0.56	-0.44	0.15	0.58	0.92	0.87	0.57	0.72	0.86	1.00																								
**Cu F3**	0.13	-0.35	0.81	-0.26	-0.10	-0.36	-0.69	-0.40	-0.33	-0.01	-0.35	-0.52	-0.20	1.00																							
**Cu F4**	0.49	0.24	-0.49	0.38	-0.55	0.24	0.65	0.57	0.62	0.48	0.23	0.70	0.49	-0.19	1.00																						
**Cu TM**	0.64	-0.30	0.47	0.05	-0.58	-0.30	-0.44	0.08	-0.02	0.09	0.24	-0.01	0.36	0.40	-0.11	1.00																					
**Ni F1**	0.57	0.10	-0.58	0.81	-0.58	0.11	0.64	0.83	0.85	0.33	0.52	0.85	0.80	-0.29	0.49	0.19	1.00																				
**Ni F2**	0.12	0.62	-0.72	0.77	-0.12	0.62	0.86	0.88	0.77	0.67	0.79	0.82	0.74	-0.56	0.53	-0.19	0.73	1.00																			
**Ni F3**	0.34	-0.35	0.73	-0.12	-0.30	-0.35	-0.70	-0.33	-0.25	0.00	-0.23	-0.44	-0.06	0.89	-0.28	0.61	-0.12	-0.51	1.00																		
**Ni F4**	0.38	0.75	-0.63	0.72	-0.40	0.75	0.65	0.41	0.51	0.78	0.46	0.47	0.37	-0.22	0.66	-0.23	0.48	0.67	-0.18	1.00																	
**Ni TM**	0.65	0.42	-0.24	0.52	-0.54	0.42	0.12	0.15	0.14	0.36	0.59	0.16	0.32	-0.16	0.01	0.53	0.44	0.27	0.16	0.42	1.00																
**ZnF1**	0.36	0.09	-0.50	0.67	-0.44	0.09	0.64	0.96	0.93	0.49	0.60	0.92	0.95	-0.29	0.51	0.21	0.88	0.79	-0.16	0.39	0.21	1.00															
**Zn F2**	0.25	0.27	-0.54	0.66	-0.28	0.27	0.74	1.00	0.88	0.55	0.73	0.92	0.93	-0.40	0.56	0.09	0.82	0.90	-0.33	0.44	0.19	0.96	1.00														
**Zn F3**	0.47	-0.48	0.48	0.00	-0.53	-0.48	-0.37	0.17	0.23	0.13	-0.06	0.10	0.43	0.63	0.08	0.69	0.23	-0.19	0.79	-0.12	0.07	0.35	0.16	1.00													
**Zn F4**	0.30	0.23	0.36	0.24	-0.29	0.23	-0.29	0.00	-0.01	0.56	0.09	-0.15	0.15	0.59	0.13	0.30	0.02	0.00	0.70	0.39	0.20	0.08	0.01	0.61	1.00												
**Zn TM**	0.48	0.07	0.50	0.10	-0.34	0.07	-0.47	-0.04	-0.20	0.24	0.37	-0.22	0.22	0.42	-0.26	0.83	0.07	-0.13	0.68	-0.04	0.66	0.03	-0.02	0.55	0.57	1.00											
**Pb F1**	0.36	0.08	-0.49	0.70	-0.44	0.09	0.62	0.95	0.92	0.48	0.58	0.90	0.94	-0.28	0.49	0.21	0.89	0.79	-0.15	0.39	0.22	1.00	0.95	0.35	0.09	0.03	1.00										
**Pb F2**	0.25	0.24	-0.65	0.48	-0.30	0.24	0.82	0.91	0.90	0.45	0.71	0.94	0.91	-0.51	0.56	0.05	0.77	0.82	-0.41	0.41	0.24	0.88	0.92	0.09	-0.16	-0.09	0.86	1.00									
**Pb F3**	0.20	-0.31	0.54	-0.30	-0.12	-0.31	-0.65	-0.33	-0.40	-0.30	-0.05	-0.41	-0.09	0.27	-0.59	0.65	-0.19	-0.49	0.60	-0.47	0.41	-0.23	-0.33	0.47	0.28	0.77	-0.22	-0.29	1.00								
**Pb F4**	-0.35	-0.74	0.75	-0.58	0.28	-0.74	-0.75	-0.46	-0.38	-0.48	-0.59	-0.55	-0.32	0.64	-0.61	0.23	-0.47	-0.72	0.58	-0.76	-0.49	-0.32	-0.48	0.48	0.09	0.09	-0.31	-0.51	0.40	1.00							
**Pb TM**	0.58	0.16	-0.17	0.33	-0.58	0.16	0.44	0.68	0.68	0.60	0.53	0.68	0.81	0.08	0.75	0.37	0.57	0.51	0.14	0.48	0.25	0.69	0.70	0.53	0.39	0.28	0.66	0.71	-0.12	-0.36	1.00						
**% Sand**	-0.32	-0.10	0.51	-0.46	0.40	-0.11	-0.72	-0.93	-0.93	-0.47	-0.62	-0.94	-0.96	0.34	-0.62	-0.16	-0.77	-0.75	0.25	-0.37	-0.15	-0.94	-0.93	-0.31	0.01	0.03	-0.91	-0.96	0.25	0.37	-0.80	1.00					
**% silt**	0.32	0.10	-0.52	0.46	-0.39	0.10	0.72	0.93	0.93	0.47	0.61	0.94	0.95	-0.34	0.62	0.15	0.77	0.75	-0.25	0.37	0.15	0.93	0.93	0.30	-0.02	-0.04	0.91	0.97	-0.26	-0.37	0.80	-1.00	1.00				
**% Clay**	0.34	0.10	-0.49	0.50	-0.41	0.11	0.69	0.94	0.93	0.50	0.62	0.94	0.97	-0.31	0.61	0.19	0.78	0.76	-0.21	0.38	0.17	0.96	0.95	0.34	0.04	0.00	0.94	0.95	-0.24	-0.36	0.81	-1.00	1.00	1.00			
**% LOI**	0.36	0.07	-0.50	0.49	-0.43	0.07	0.69	0.92	0.93	0.45	0.63	0.93	0.97	-0.31	0.57	0.23	0.81	0.73	-0.21	0.34	0.22	0.95	0.93	0.32	-0.04	0.01	0.93	0.96	-0.22	-0.34	0.78	-0.99	0.99	0.99	1.00		
**NO_3 (ug/L)**	-0.33	-0.78	0.73	-0.77	0.26	-0.79	-0.81	-0.65	-0.60	-0.69	-0.77	-0.66	-0.56	0.48	-0.58	0.16	-0.67	-0.88	0.41	-0.85	-0.53	-0.56	-0.68	0.29	-0.09	-0.01	-0.55	-0.67	0.42	0.90	-0.51	0.56	-0.56	-0.56	-0.55	1.00	
**NH_4 (ug/L)**	0.32	0.33	-0.53	0.90	-0.34	0.33	0.60	0.88	0.74	0.56	0.60	0.76	0.75	-0.34	0.44	0.06	0.84	0.86	-0.23	0.54	0.28	0.87	0.87	0.11	0.16	0.03	0.89	0.66	-0.32	-0.49	0.45	-0.69	0.68	0.72	0.69	-0.69	1.00	
**TN (ug/L)**	-0.10	-0.84	0.47	-0.38	-0.01	-0.84	-0.44	0.03	-0.04	-0.45	-0.48	-0.03	0.03	0.27	-0.15	0.28	-0.12	-0.38	0.23	-0.69	-0.57	0.11	-0.02	0.52	-0.09	-0.08	0.11	-0.12	0.20	0.70	-0.06	-0.06	0.06	0.08	0.06	0.72	-0.07	1.00

The abbreviation used in the table are : DO = Dissolved Oxygen (mg/L); ORP= Oxidation Reduction Potential (mV); TDS= Total Dissolved Solid (ppm); Temp.=Temperature; Cr=Chromium; Cu=Copper; Ni= Nickel; Zn= Zinc; Pb= Lead; F1= Fraction 1 (exchangeable and bound to carbonates); F2= Fraction 2 (bound to iron and manganese oxide); F3= Fraction 3 (bound to organic matter and sulphides); F4= Fraction 4 (residual fraction); TM= Total Metal; %LOI= Loss of Ignition %; TN= Total Nit

Table S4. Nitrogen content of the overlying water, LOI% of the sediments and population (density/km[2]) of the studied sampling sites.

Site no.	$NO_3 + NO_2$ (ug/L)	NH_4 (ug/L)	TN[1](ug/L)	LOI % [2](organic matter)	Population (Density/km[2])
S1	260	<2	280	0.4	5
S2	360	<2	390	0.2	5
S3	250	<2	280	0.4	5
S4	270	<2	290	0.2	28
S5	250	21	360	0.2	28
S6	170	5	220	0.4	392
S7	23	37	110	0.2	370
S8	69	110	310	1.8	1313
S9	230	6	280	0.4	676
S10	110	24	270	1.8	896

[1]Total Nitrogen; [2]Loss on Ignition

Table S5. Risk Assessment Code (RAC %) values of Cu, Ni, Zn and Pb at all sampling sites.

Site	Cu	Ni	Zn	Pb
S1	0	3.9	2.5	0
S2	1.1	2.2	2.6	0
S3	0	2.9	2.1	0
S4	0	2.9	1.8	0
S5	1.7	3.9	2.4	0
S6	4.8	4.7	3.3	0
S7	2.5	2.4	2.1	0
S8	8.8	5.9	6.8	5.2
S9	3.5	5.3	4.2	0
S10	7.8	4.4	5.1	2.6

References:

[19] Singh, S.K., Sarin, M.M., France-Lanord, C. Chemical erosion in the eastern Himalaya: Major ion composition of the Brahmaputra and δ13C of dissolved inorganic carbon. *Geochim. Cosmochim. Acta.* **2005**, *69*, 3573–3588. https://doi.org/10.1016/j.gca.2005.02.033

Population density source: https://www.census2011.co.in

© 2020 by the authors. Licensee MDPI, Basel, Switzerland. This article is an open access article distributed under the terms and conditions of the Creative Commons Attribution (CC BY) license (http://creativecommons.org/licenses/by/4.0/).
